# Self-assembled DNA nanocarrier-enabled drug delivery for bone remodeling and antimicrobial applications

**DOI:** 10.1038/s44385-025-00020-2

**Published:** 2025-09-03

**Authors:** Jiaqi Huang, Aishik Chakraborty, Yasmeen Shamiya, Wei Luo, Alap Ali Zahid, Arghya Paul

**Affiliations:** 1 Department of Chemical and Biochemical Engineering, The University of Western Ontario, London, ON N6A 5B9 Canada; 2 Collaborative Specialization in Musculoskeletal Health Research and Bone and Joint Institute, The University of Western Ontario, London, ON N6A 5B9 Canada; 3 Department of Chemistry, The University of Western Ontario, London, ON N6A 5B9 Canada; 4 School of Biomedical Engineering, The University of Western Ontario, London, ON N6A 5B9 Canada

**Keywords:** Nanoparticles, Biomedical materials, DNA and RNA, Drug delivery, Bacterial infection, Bone

## Abstract

We report a synthetic tetrahedral DNA nanocarrier (TDN) for treating bone defects and methicillin-resistant *Staphylococcus aureus* (MRSA) infection using in vitro studies. We successfully synthesized TDNs and demonstrated their excellent cytocompatibility with blood cells and immune cells. Zoledronic acid-loaded TDN displayed increased efficacy compared to free drugs in regulating bone remodeling, while vancomycin-loaded TDN showed an increased antibacterial effect against MRSA. In the future, this nano-drug delivery platform, with its multifunctional properties, can be potentially used to treat bone fractures and surgery-related MRSA infection.

Bone fracture is a significant public health concern, particularly for older adults. Up to 37 million fractures occur annually in individuals aged over 55, equivalent to 70 fractures per minute^1^. One of the major risk factors for bone fractures is osteoporosis, characterized by reduced bone mineral density. The International Osteoporosis Foundation reported that the prevalence of osteoporosis fractures in people aged over 50 was estimated to be around 1 in 3 women and 1 in 5 men^2^. The cellular mechanisms underlying osteoporosis involve the bone remodeling mediated by osteoblasts and osteoclasts^3^. Osteoblasts are responsible for bone formation, while osteoclasts mediate bone resorption. The pathology of osteoporosis primarily stems from an imbalance in the bone remodeling process, where bone resorption by osteoclasts outpaces bone formation by osteoblasts^4^.

The current widely recognized first-line drug treatment for osteoporosis is zoledronic acid (ZA), an antiresorptive medication that belongs to the third generation of bisphosphonate drug class^5^. ZA reduces bone resorption by inhibiting osteoclast proliferation and inducing osteoclast apoptosis^6^. However, direct ZA administration by intravenous injection is accompanied by a range of dose-related side effects and reduced efficacy after long-term use. The most observed side effect involves the acute phase reaction characterized by fever, fatigue, aches, and pains^7^. ZA can also lead to more serious but less common adverse effects, including jaw osteonecrosis and severe inflammation^8^. To overcome these adverse effects, it is imperative to design more effective drug delivery methods.

Osteoporosis also poses significant challenges in orthopedic surgery if a bone fracture occurs, particularly concerning the risk of Methicillin-resistant *Staphylococcus aureus* (MRSA) infection^9^. Direct administration of vancomycin remains the first-line treatment for MRSA infections. However, the emergence of vancomycin tolerance and adverse effects, including nephrotoxicity, nausea, and vomiting, poses significant challenges to its effectiveness^10,11^. Studies have documented an incremental increase in the MIC of vancomycin in MRSA isolates, which correlates with higher clinical failure rates^12^. As such, advanced drug delivery methods to increase drug efficacy need to be established.

Nanoparticles have emerged as innovative drug delivery systems, offering numerous advantages over the traditional direct administration of free drugs. One of the primary advantages of using nanoparticles for drug delivery is the improvement of the solubility and stability of poorly soluble drugs, and providing sustained and controlled drug release^13^. Encapsulation of these drugs by nanoparticles enhances the bioavailability, pharmacokinetics, and therapeutic efficacy compared to free drug formulations^14^. Although organic nanomaterials such as liposomes and cationic polymers could increase the intracellular uptake and prolong the in vivo half-life of the drugs, their complex synthesis mechanisms and lack of programmability hinder their development toward clinical applications^15^. Inorganic nanoparticles, such as gold nanoparticles and metal oxide nanoparticles, are highly flexible, enabling functional modification and controllable drug release^16^. Nevertheless, these nanoparticles are not free from toxicity concerns and biodegradation challenges^17^.

Tetrahedral DNA nanocarriers (TDNs) have been shown to have promising potential as an alternative vehicle for drug delivery and biomedical treatment^18^. In addition to the existing advantage of nanoparticles in drug delivery, TDNs were also proven to have excellent biocompatibility, better cellular internalization, and functional versatility^19^. Through controllable self-assembly from 4 different DNA strands and potential modifications, TDNs demonstrated exceptional programmability in delivering different therapeutic agents^20^. Recent studies have demonstrated that ampicillin-loaded TDN overcame antibiotic resistance in MRSA^21^ and promoted osteogenic differentiation in stem cells^22^. In this brief communication, we designed the TDN and synthesized ZA-loaded TDNs (Z-TDN) and vancomycin-loaded TDNs (V-TDN) as nanomedicine for treating osteoporosis and related MRSA infection in orthopedic surgery.

TDNs were synthesized by temperature-controlled self-assembly from 4 artificially designed single-strand DNAs (ssDNA) as shown in Fig. 1a and Supplementary Fig. 1. The molecular weight of each ssDNA is 63 bp, and by calculation, we hypothesize that the fabricated TDN is 252 bp with an 8–10 nm length on each side. Agarose gel electrophoresis displayed the relative molecular weight of ssDNA, partially assembled DNA structures, and TDNs, which is consistent with the calculation (Fig. 1b). Transmission electron microscopy (TEM) was employed to verify the triangular shape of TDNs as marked in the red dotted lines (Fig. 1c). These data collectively demonstrated the successful assembly of TDNs.

**Fig. 1:**
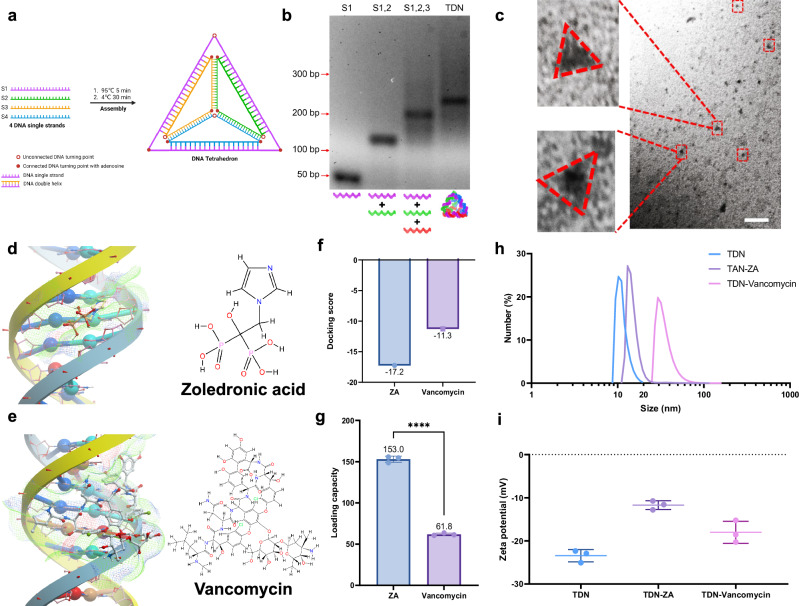
Preparation and characterization of TDN, Z-TDN, and V-TDN. **a** Schematic shows the synthesis of TDNs via temperature-mediated self-assembly. **b** Agarose gel electrophoresis confirms the formation of TDNs. **c** TEM images display the shape and size of TDNs (red dotted lines). Scale bar = 100 nm. 3D demonstration of single molecule interaction between **d** TDNs and ZA and **e** TDNs and vancomycin using molecular simulation. **f** The graph displays the interaction between ZA/vancomycin and TDN represented by the docking score. A lower binding score suggests higher affinity. **g** Experimental drug loading capacity of TDN for ZA and vancomycin. **h** DLS shows the size distribution of TDN, Z-TDN, and V-TDN. **i** The figure shows the zeta potential of TDN, Z-TDN, and V-TDN.

Next, we used an emerging method, molecular modeling, to predict the capacity of TDN to load ZA and vancomycin^23^. Molecular docking simulations showed that the most stable loading position of ZA was in the DNA minor groove (Fig. 1d) while vancomycin can only fit in the DNA major groove due to larger molecule size (Fig. 1e). As a result, the binding affinity of TDN towards ZA is higher than vancomycin (Fig. 1f). Consistent with the simulation, experimental drug-loading performed by direct incubation showed TDN has a significantly higher loading capacity of ZA compared to vancomycin (Fig. 1g). We also measured particle size and zeta potential of TDN, Z-TDN and V-TDN. Dynamic light scattering (DLS) showed an increase in particle size with V-TDN (~29 nm) compared to TDN (~10 nm) and Z-TDN (~14 nm). We observed higher zeta potential with Z-TDN (-11 mV) compared to TDN (-23 mV) and V-TDN (-18 mV). The increase in particle size and zeta potential also indicates successful drug-loading.

Both ZA and vancomycin are FDA-approved drugs for clinical treatment, but as a new biomedical platform, TDNs require biosafety examination before potential clinical applications. Multiple blood-related cells were selected to test the biocompatibility of TDN. Human monocytic cells (THP-1) were incubated with TDNs for 24 h followed by an MTS cell proliferation assay (Fig. 2a) and Calcein staining (Fig. 2b). MTS cell proliferation assay showed no significant cytotoxic effect of TDNs on THP-1 cells with concentrations from 0.1 to 1 μM. Calcein staining of cells treated with the corresponding concentrations of TDN also displayed cells with healthy morphology, similar to the untreated control group. To further investigate the biocompatibility of TDNs, we observed their hemolytic effects using safety guidelines established by the FDA protocol ASTM-F756^24^. The assay showed 0.1 μM TDN induced 2.1% hemolysis, which is considered non-hemolytic. Higher concentrations of TDN at 0.5 μM and 1 μM were tested hemolytic, showing 9.8% and 16.5% hemolysis, respectively (Fig. 2c). Corresponding images of red blood cells incubated with TDNs and Triton-X (positive control with 100% hemolysis) showed the expected morphology (Supplementary Fig. 2). Additionally, the immune-related activity of TDN was examined for its apoptotic, genotoxic, and inflammatory effects. An apoptosis assay (Annexin V and PI staining) displayed no significant increase in apoptotic cells after 24 h TDN exposure to THP-1 cells (Fig. 2d). Next, an alkaline comet assay was used to examine the genotoxicity of TDN when exposed to human mesenchymal stem cells (hMSCs) for 24 h. Compared to the positive control (NiO) which showed a mean of 17.5% tail DNA, cells exposed to the TDN group showed little genotoxicity (4.79% tail DNA) and closely resembled the negative control (4.95% tail DNA) with a comet head and no observable comet tail, as indicated in the representative images (Fig. 2f, g). To test inflammatory activity, human peripheral blood mononuclear cells (hPBMCs) were incubated with TDNs for 16 h, and we used the well-known inflammatory stimulant CpG DNA as a positive control. ELISA results showed a significant increase in IL-6 and TNF-α secretion of hPBMCs in TDN-CpG at 1 μM but no significant increase in cells exposed to TDNs at varying concentrations (Fig. 2h, i). From in vitro biosafety assays, we conclude that TDN has excellent biocompatibility and that 0.1 μM is the best concentration for further testing using in vitro bone remodeling and MRSA infection models.

**Fig. 2:**
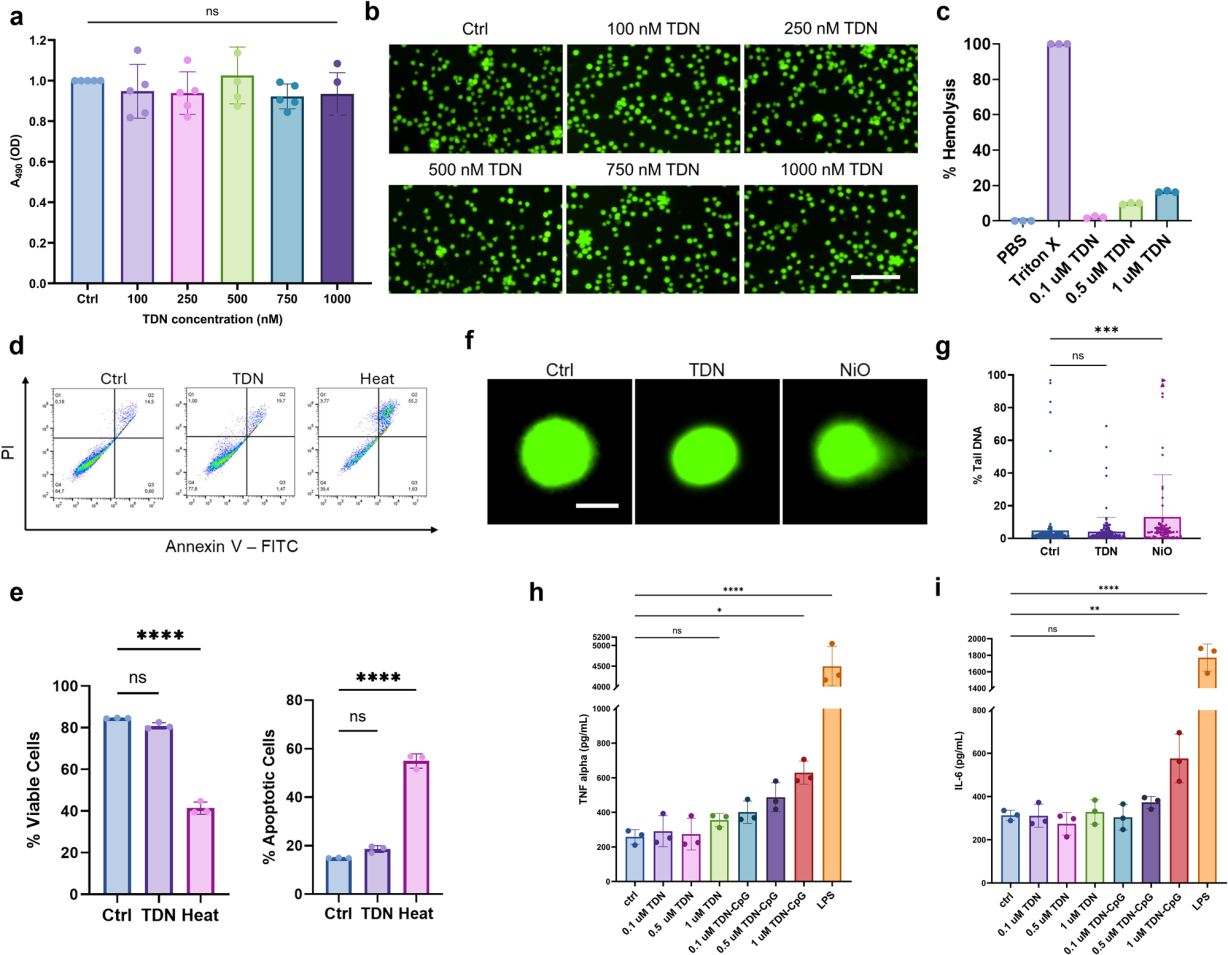
In vitro cytocompatibility of TDN. **a** THP-1 cell (a human monocytic cell line) proliferation was analyzed by MTS assay after 24 h incubation with different concentrations of TDN, showing no cytotoxicity (*n* = 5). **b** Corresponding Calcein AM fluorescence images of THP-1 cells after 24 h incubation with TDNs. Scale bar = 100 μm. **c** Percentage of hemolysis induced by 0.1, 0.5, and 1 μM TDN with PBS as a negative control and Triton-X as a positive control (*n* = 3). 0.1 μΜ TDN is non-hemolytic. **d**, **e** Annexin V/PI staining profiles of THP-1 after 24 h exposure to treatment groups show no significant increase in apoptosis when cells are exposed to TDN (*n* = 3). No significant increase in apoptosis when cells are exposed to TDNs. (**f**, **g**) % Tail DNA measurement (**f**) and representative images (**g**) of the alkaline comet assay assessing DNA damage of human mesenchymal stem cells (hMSCs) exposed to NiO (positive control), TDNs, or left untreated (negative control) for 24 h (*n* = 3). hMSCs exposed to TDNs show little to no genotoxicity, whereas NiO-treated cells showed increased genotoxicity, as concluded by the presence of a comet tail. Scale bar = 25 μm. **h**, **i** Graph shows ELISA analyses of the inflammatory cytokines TNF-α (**h**) and IL-6 (**i**) secreted by human peripheral blood mononuclear cells (hPBMCs) after 16 h stimulation with TDNs, TDNs with CpG, and LPS (positive control). No significant inflammatory response was observed for TDNs (*n* = 3).

The effect of Z-TDN on bone remodeling was examined by osteoblast and osteoclast differentiation assays. In the osteogenesis experiment, Alizarin Red Staining (ARS) was performed as a visualization of calcium deposits (Fig. 3a). The staining showed increased mineralization of 0.5 μM Z-TDN compared to 0.5 μM free ZA. Next, ARS dye was extracted and quantified by measuring the absorbance at 405 nm (Fig. 3b). ARS quantification displayed significantly increased osteoblast differentiation with Z-TDN when compared to free ZA. Additionally, we focused on the effect of ZA and Z-TDN on osteoclast differentiation (Fig. 3c). Tartrate-resistant acid phosphatase (TRAP) assay was performed to visualize and count osteoclast cells. TRAP staining displayed reduced osteoclast cell numbers in Z-TDN compared to ZA. Quantification of osteoclast cell number showed significantly better inhibition of osteoclast differentiation by Z-TDN than free ZA at the same concentration.

**Fig. 3:**
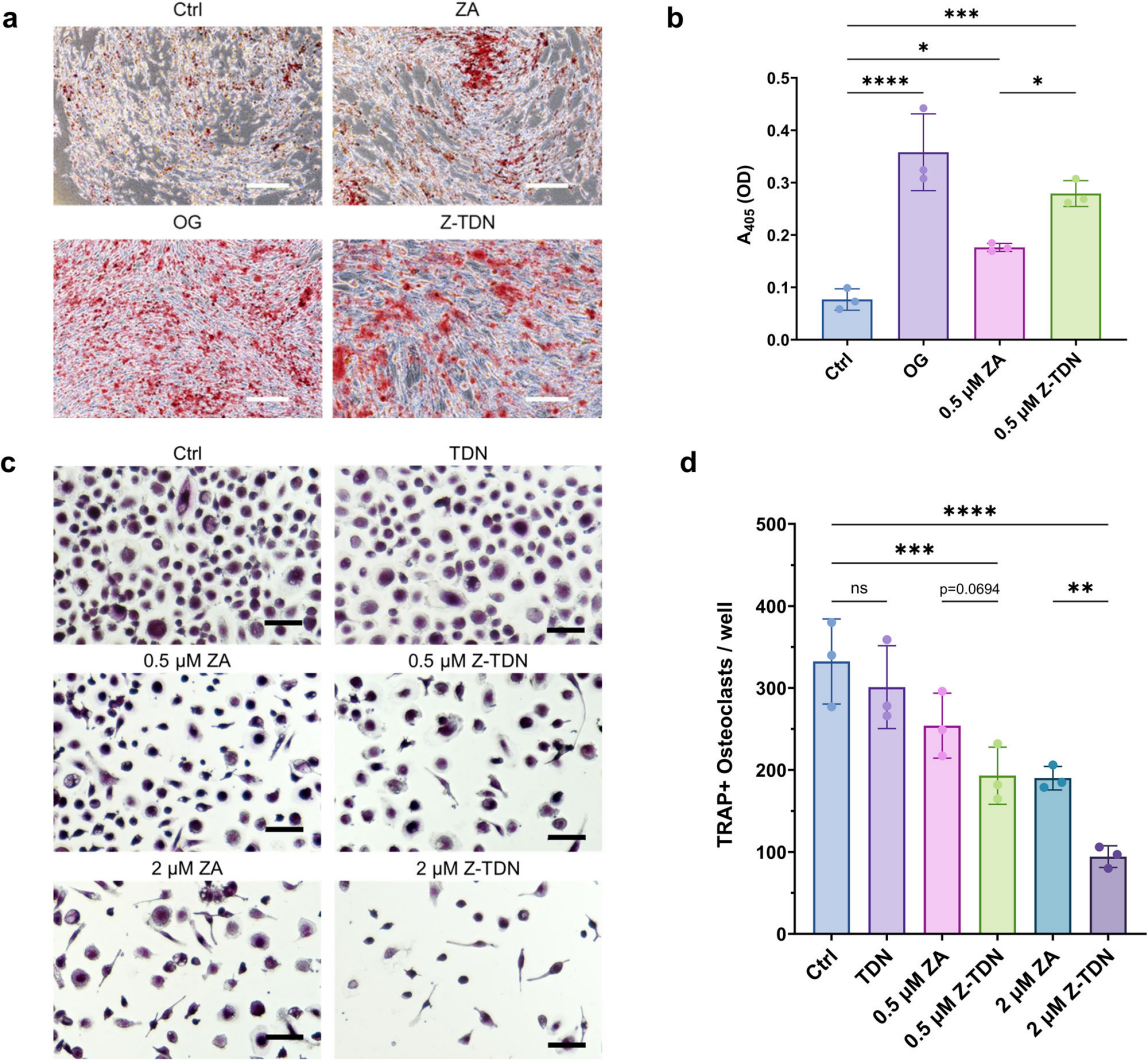
In vitro regulatory effect of Z-TDN on bone remodeling. **a** Microscopy images of calcium deposits stained by Alizarin Red Staining (ARS) after osteoclast differentiation experiment. Pre-osteoblasts were treated with osteogenic media (positive control), ZA, Z-TDN, or left untreated. Z-TDN displayed increased mineralization compared to free ZA (*n* = 3). Scale bar = 500 μm. **b** Quantification of extracted ARS showed significantly increased osteogenic differentiation with Z-TDN compared to free ZA. **c** Microscopy images of tartrate-resistant acid phosphatase (TRAP) staining on osteoclasts treated with TDN, ZA, Z-TDN, or left untreated. Z-TDN group displayed reduced TRAP-positive cells and smaller cell size compared to free ZA. Scale bar = 100 μm. **d** Corresponding osteoclast count per well showed enhanced inhibition of osteoclast activity of Z-TDN compared to free ZA (*n* = 3).

After confirming the increased efficacy of ZA by Z-TDN on inhibition of bone remodeling, we aimed to deliver vancomycin using V-TDN to inhibit MRSA growth. The minimum inhibitory concentration (MIC) of vancomycin on MRSA was determined as 4 μg/mL following the standard microdilution method^25^ (Supplementary Fig. 3). Next, the antibacterial assay was performed using free vancomycin and the same concentration in V-TDN form. After 24 h of incubation, 0.75 MIC V-TDN showed a significant antibacterial effect compared to 0.75 MIC free vancomycin (Fig. 4a). Bacterial growth curve also showed increased efficacy against MRSA by V-TDN and displayed a delayed MRSA growth (Fig. 4b). The corresponding bacteria in each group were streaked onto the plate, and it can be clearly observed that the 0.75 MIC V-TDN group has fewer MRSA colonies than 0.75 MIC vancomycin. Collectively, the TDN-based delivery method reduced the dosage of vancomycin by 25%, demonstrating enhanced drug efficacy and the clinical potential for overcoming antibiotic resistance.

**Fig. 4:**
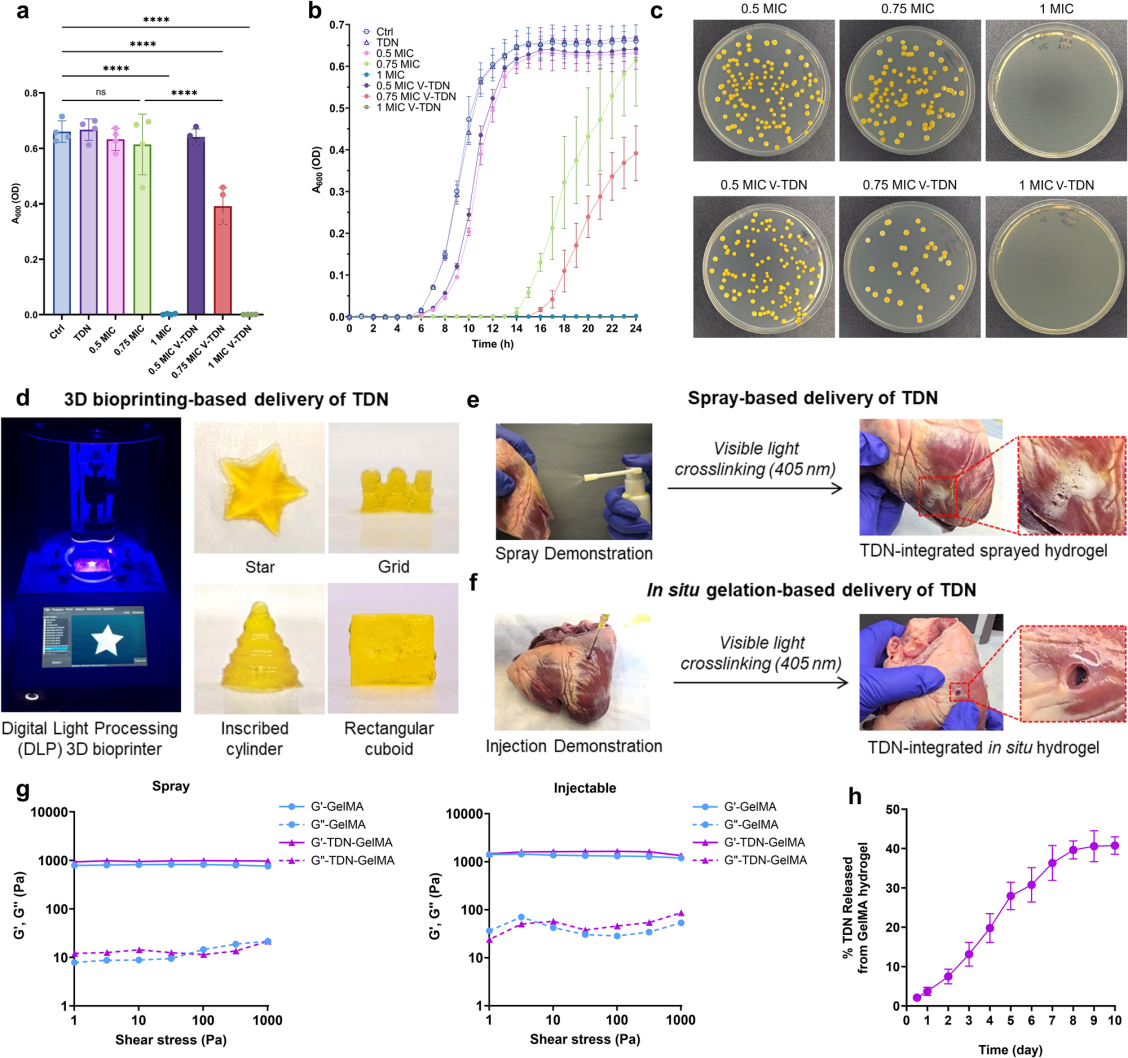
In vitro antimicrobial effect of V-TDN and potential scaffold-embedded delivery methods. **a** The plot demonstrates OD_600_ of MRSA after 24 h exposure to 0.5, 0.75, or 1 MIC vancomycin, V-TDN, or left untreated. 0.75 MIC V-TDN showed a significant antibacterial effect compared to 0.75 MIC free vancomycin (*n* = 4). **b** Effects of free vancomycin and V-TDN on the growth of MRSA cells. 0.75 MIC V-TDN showed enhanced inhibition of MRSA growth compared to free vancomycin at the same concentration. **c** Corresponding pictures of MRSA colonies on TS-agar plates after 24 h. The reduced number of MRSA colonies indicated that V-TDN increased the efficacy of vancomycin against MRSA. **d** Image shows a digital light processing (DLP) 3D bioprinter-based scaffolding platform for delivering TDNs. DLP allows for fast and high-resolution bioprinting of TDN-integrated GelMA hydrogel. Multiple bioprinted shapes have been displayed here to demonstrate the versatility of the nanocomposite material and the bioprinting platform. **e**, **f** Crosslinking of sprayed (**e**) and injected (**f**) TDN-integrated GelMA hydrogel by a blue light on the porcine heart surface. **g** Representative stress sweeps of spray and injectable GelMA or TDN-integrated GelMA hydrogels through multiple delivery methods. The results demonstrate a very similar rheological profile between GelMA and TDN-integrated GelMA hydrogels. **h** The cumulative drug release profiles of 1 μM TDN-integrated GelMA hydrogel in PBS over 10 days.

TDNs have been traditionally delivered mainly by intravenous injection^26^ or subcutaneous injection^27^. In recent studies, TDNs were delivered by nasal administration^28,29^. We explored alternative delivery methods for TDNs tailored for surgical-related applications and bone regeneration. TDNs can be integrated within gelatin methacryloyl (GelMA) hydrogel and 3D-bioprinted to be delivered as implants, which can conform to personalized designs (Fig. 4d). We demonstrated 3D bioprinting of TDN-integrated hydrogel in multiple shapes, including star, grid, inscribed cylinder, and rectangular cuboid. Additionally, the porcine heart was used as a model tissue for the development of spray-based and injection-based delivery of TDN-integrated hydrogels. We formed TDN-integrated in situ hydrogel using visible light crosslinking on the porcine heart after being sprayed or injected (Fig. 4e, f and Supplementary Movie 1–5). The feasibility of using injection-based and spray-based TDN-integrated hydrogel formulations was demonstrated by assessing the rheological properties, which showed very similar rheological profiles compared to the GelMA hydrogel (Fig. 4g). In addition, a drug release study displayed a steady release of TDN from GelMA hydrogel, with ~40% release in 8 days before plateauing (Fig. 4h). These novel TDN-based delivery methods broaden the potential clinical applications of TDNs for localized specific delivery in bone surgery and antibacterial therapy.

Our work established TDNs as an innovative DNA-based nanoplatform for the efficient delivery of ZA and vancomycin. Z-TDN showed an approximately 3-fold increased drug efficacy in promoting osteogenic differentiation and inhibiting osteoclast activity compared to the current free drug formulation, which is comparable to other emerging nanomedicines^29,30^. We also demonstrated that TDN-based drug delivery could increase the antibacterial effect of vancomycin in treating MRSA. Finally, we explored additional delivery methods, including 3D bioprinted hydrogels and TDN-integrated in situ hydrogel by spray or injection. Our research broadened the application with more flexible delivery approaches of TDN, facilitating the osteogenic and antibacterial application of TDN in bone surgery. Future investigations can focus on using in vivo osteoporotic and MRSA-infected animal models^31^. Finally, we envision a combined therapy to co-deliver V-TDN and Z-TDN in the prevention of bone fractures, cancer bone metastases, Paget’s disease, osteopenia, and associated infections.

## Methods

### Materials

Zoledronic acid (PHR1893), melatonin (PHR1767), Dulbecco’s Modified Eagle’s Medium (DMEM) (D5796), Dulbecco′s Phosphate Buffered Saline (PBS) (D8537), and ultrapure Nuclease-free water (W4502) were purchased from Sigma-Aldrich. Ultrapure 1 M Tris-HCl (J22638), Magnesium chloride hexahydrate (213832), 50 bp DNA Ladder (10416014), and TrackIt™ Cyan/Orange Loading Buffer (10482028) were purchased from Thermo Fisher Scientific. Powder-formed ssDNA was purchased from Thermo Fisher Scientific oligonucleotide synthesis service with the sequence shown in Table 1.

**Table 1:** Specific sequence of each ssDNA

Name	Sequence (5’ → 3’)	GC content	Molecular weight (g/mole)
S1	ACTTTACATCCGCCATAGTAGACGTATCACCAGGCACTTGAGACGGTCATTCCTAAGTCTGAA	48%	19,310
S2	AGAAGCGATGCAACTCTACCGACGATTAACGCTTGCTACACGATTCAGACTTAGGAATGACCG	48%	19,393
S3	ACTACTATGGCGGATGTAAAGACGTGTAGCAAGCGTTAATCGACGGTTAGAGCATGCCCATCC	49%	19,480
S4	ACGGTAGAGTTGCATCGCTTCACTCAAGTGCCTGGTGATACGAGGATGGGCATGCTCTAACCG	52%	19,479

### Agarose gel electrophoresis

Agarose gel electrophoresis was run with 2% agarose gel (1613100, Bio-Rad) at 120 V in TAE buffer for 60 min. After electrophoresis, the bands of DNA were stained with 1× SYBR Safe and the image was visualized using ChemiDoc XRS+ Molecular Imager and processed using Image Lab Software (Bio-Rad).

### Transmission electron microscopy (TEM)

Two micrometer TDNs were synthesized and subsequently fixed using a 2% glutaraldehyde solution before TEM. TDNs were stained with phosphotungstic acid (PTA) and imaged with CM10 TEM (PHILIPS) with a maximum magnification of 13,000×.

### Molecular simulation

ICM-Pro was utilized for the simulation of molecular interactions between TDN and small-molecule drugs, including ZA and vancomycin. The 3D molecular model of ZA and vancomycin was obtained from PubChem. The molecular docking simulation was performed by treating linear dsDNA or TDN as a receptor and ZA or vancomycin as a ligand. The docking score was calculated by the ICM Pro software. For each simulation, 100 top hits with lower binding energy scores were generated, and the result with the highest affinity was selected. Each simulation was repeated 5 times.

### Preparation of drug-loaded TDNs and the measurement of drug-loading capacity

Drug-loading was conducted using an established protocol^32^. 100 μL of 2 μM DNA TDN was prepared 24 h before the experiment and then mixed with 100 μL of 1 mM ZA or vancomycin. The final concentration was 1 μM TDN and 500 μM free drug. The mixture was incubated at 37 °C, 60 rpm for 3 h to make sure each drug was sufficiently intercalated into TDNs before the miscible liquids were passed through Amicon ultracentrifuge filters by centrifuging at 14,000*g* for 10 min. Next, the unloaded drug concentration was measured by UV-Vis absorbance at 209 nm for ZA and 280 nm for vancomycin using a SPARK microplate multimode reader (Tecan). The drug-loading capacity was calculated by the following formula:

$$\text{Drug}-\text{loading}\,\text{capacity}=\,\frac{\left[\text{Drug}\,\text{added}\right]-\left[\text{Unload}\,\text{drug}\right]}{[\text{TDN}]}$$

### Cell viability and proliferation studies

Cell viability and cell proliferation studies were performed by Calcein AM staining (Thermo Fisher Scientific, cat. No. C1430) and MTS assay (Promega, Cat. No. G3580). THP-1 monocytic cells were seeded into 96-well plates with a seeding density of 1 × 10^5^ per well and cultured for 12 h. Fresh media were added, and the cells were treated with 100, 250, 500, 750, and 1000 nM TDN. All the experiments were performed following the manufacturer’s protocol, and the live cell images were taken using the fluorescent Nikon Eclipse Ti2-E microscope (Nikon).

### Hemolysis studies

Mouse red blood cells (mRBCs) were isolated from whole blood samples purchased from Innovative Research. The mRBCs were separated from the serum and washed three times with sterile DPBS by centrifugation at 3460 rpm for 10 min at 4 °C. Next, mRBCs were diluted with DPBS to a final concentration of 0.16% (v/v) mRBCs suspension. Then, 0.16% mRBCs suspension was incubated with DPBS, 100 nM TDNs, 500 nM TDNs, 1000 nM TDNs, and with 0.2% Triton-X as a positive control. The plate was incubated at 37 °C with agitation for 1 h, after which the supernatant was transferred to Eppendorf tubes and centrifuged at 14,000 rpm at 4 °C for 10 min to remove any intact cells. Hemolytic activity was assessed by measuring the amount of hemoglobin liberated into the surrounding solution due to membrane rupture. The amount of hemoglobin released was determined by measuring the absorbance at 415 nm. Controls defining 0 and 100% hemolysis were plated in DPBS in the absence or presence of 0.2% Triton-X, respectively. Representative images of mRBCs in the supernatant were taken using a Nikon Eclipse Ti2-E microscope.

### Cell apoptosis study in the presence of TDNs

Cellular apoptosis was determined using Annexin V and a propidium iodide staining kit (Biotium, Cat. No. 30061). In brief, THP-1 cells were seeded into a six-well plate in suspension at a density of 1 × 10^5^ per well in a 96-well plate and incubated for 24 h. The next day, the cells were treated for 24 h with TDNs (500 nM) and CPG-TDNs (500 nM). The positive control was made by exposing the cells to 55°C for 20 min, followed by overnight incubation at 37 °C in a CO_2_ incubator^14^. All the samples were stained according to the manufacturer’s specified protocol and analyzed using the Cytoflex flow cytometer (Beckman Coulter).

### Cell genotoxicity assessment using alkaline comet assay in the presence of TDNs

The alkaline comet assay was carried out according to the manufacturer’s protocol (R&D Systems, Cat. No. 4250-050-ESK). Briefly, human mesenchymal stem cells (hMSCs) purchased from Lonza were seeded in a 48-well plate with a seeding density of 30,000 cells/well and cultured overnight. The next morning, cell media was replaced with fresh media and was either left untreated (negative control) or treated with 30 µg/mL NiO (positive control) or 0.5 µM TDNs and exposed for 24 h. Cells were then harvested by trypsinization and resuspended in cold PBS. 50 µL of the cell suspension was added to 500 µL of LMAgarose. 50 µL of the resulting cell/gel solution was pipetted onto CometSlides. Cells were lysed for 1 h in the dark and placed in an alkaline buffer for unwinding. This was followed by electrophoresis at 25 V for 30 min in an alkaline buffer. Samples were then washed, dried overnight, and fixed in methanol. DNA was stained using 1:10,000 SYBR-Green in TAE buffer for 15 min. DNA was imaged using a fluorescence microscope (Nikon) and scored using OpenComet ImageJ software. A minimum of 30 comets were scored per sample, and results were expressed as a mean of percent of DNA in tail ± SD. Three individual experiments were performed for each group.

### Inflammation studies with hPBMCs and TDNs

Human peripheral blood mononuclear cells (hPBMCs) were purchased from Lonza. Cells were cultured in a T-75 flask for 6 days in RPMI medium with 10% FBS, 100 U/ml penicillin, 2 mM l-glutamine, and 25 ng/mL M-CSF for macrophage differentiation. hPBMCs were seeded in a 96-well plate at a density of 5 × 10^4^ cells and incubated overnight. PBS, 100, 500, or 1000 nM TDNs were added to the cells, and 1 µg/ml lipopolysaccharides (LPS) (Sigma Aldrich, Cat. No. L6529-1MG) were added separately as a positive control. Supernatants collected at 1 h after hPBMC seeding without differentiation media were used as negative controls. After 6 h, the supernatant was collected, and TNF-α and IL-6 secretion were quantified using the Quantikine ELISA Kit (R&D Biosystems) according to the manufacturer’s protocol.

### In vitro osteogenic differentiation and osteoclast inhibition assay

Human MSCs were cultured on 100 mm petri dishes in stem cell culture media until reaching 100% confluency. hMSCs were seeded in a 48-well plate at a density of 3 × 10^4^ cells/well in hMSC media supplemented with 5% FBS and 1% PS. Upon reaching 100% confluency, 0.5 μM ZA or 0.5 μM Z-TDN was added or replaced by osteogenic media supplemented with 10 mM BGP and 0.5 mM l-ascorbic acid (Sigma). After 14 days, ARS staining was performed according to the manufacturer’s protocol (ScienCell). hPBMCs were differentiated into macrophages for 6 days using 20 ng/mL M-CSF and then seeded onto a 96-well plate at a density of 2 × 10^4^ per well. Seeded macrophages were differentiated into osteoclasts using 20 ng/mL M-CSF and RANK-L for 6 days. Next, the media was replaced with LGM media (Lonza) and treated with ZA, Z-TDN, or left untreated for 3 days. Osteoclast activity was visualized by TRAP staining according to the manufacturer’s protocol^33^ (Sigma). TRAP-positive osteoclasts were counted using OpenCFU version 3.9.

### Antibacterial activity of V-TDN

Antibacterial assay of V-TDN was performed following the previous publication^34^. Overnight cultures of MRSA were grown in TSB medium at 37°C with shaking. Cultures were pelleted at 13,000*g* for 3 min, washed twice with DPBS, and resuspended in DPBS. The optical density at 600 nm (OD_600_) of the bacterial suspension in each group was determined, and the cells were normalized to an OD_600_ equal to 0.1 in DPBS. A 0.2 mL suspension of TSB and the experimental group was inoculated with MRSA at 37 °C in a 96-well plate with shaking for 24 h. The OD_600_ values of bacterial cultures were measured before diluting and plating onto TS-agar to enumerate CFUs at 24 h post-inoculation.

### Fabrication of 3D bioprinted, injectable, and sprayable TDN-integrated GelMA hydrogel

#### 3D bioprinted scaffold

To prepare 3D bioprintable TDN-integrated GelMA hydrogels, GelMA prepolymer (15% w/v), lithium phenyl-2,4,6-trimethylbenzoylphosphinate (1% w/v), Tartrazine (0.1% w/v), and 0.5 μM TDNs were dissolved in PBS at 70 °C. TDN-integrated GelMA pre-gel solution was 3D bioprinted using a Lumen X DLP 3D printer (CELLINK) following an established protocol^35^.

#### Injectable scaffold

To prepare TDN-integrated GelMA pre-gel solution for in situ gelation, GelMA prepolymer (5% w/v), lithium phenyl-2,4,6-trimethylbenzoylphophinate (1% w/v), Tartrazine (0.1%, w/v) and 1 μM TDNs were dissolved in DPBS at 70 °C. A 6 mm biopsy punch was made on the surface of a porcine heart. 300 µL of TDN-integrated GelMA pre-gel solution was injected with a 1 ml syringe, and the solution was crosslinked with a 405 nm blue light for 10 min.

#### Sprayable scaffold

To prepare a TDN-integrated GelMA sprayable scaffold, the same hydrogel pre-gel solution preparation procedure was performed as in situ gelation. The pre-gel solution was sprayed using a 10 mL spray bottle onto the surface of a porcine heart and exposed to a 405 nm blue light for 10 min.

### Characterization of sprayable and injectable TDN-integrated GelMA hydrogel

#### Rheological analysis

Rheological analysis was performed using a HAAKE Modular Advanced Rheometer System (MARS) (Fisher Scientific). Sprayed and injected TDN-integrated GelMA hydrogels or GelMA hydrogels with the same reparation method were tested using a P20/Ti titanium plate. Stress sweep tests were performed at 37 °C from 0.1 to 10^4^ Pa^34^.

#### TDN release study

TDN-integrated GelMA (15% w/v) hydrogels were prepared and printed into cylinders with a volume of 0.2 mL. TDN-integrated GelMA hydrogels were then incubated at physiological conditions (1.5 mL of PBS at 37 °C on an orbital shaker) for 10 days until a plateau was reached. TDN release was measured by the absorbance of the supernatant at 260 nm and percentage release was calculated by blanking with the measurements of GelMA hydrogels in the same conditions.

### Statistical analysis

The variations among the experimental groups were quantified using ordinary one-way ANOVA with Tukey post hoc comparisons. GraphPad Prism 10 was used for all statistical analyses. All the experimental data were calculated as mean ± standard deviation. Values of *p* < 0.05 were considered to be statistically significant (*p < 0.05, **p < 0.01, ***p < 0.001, ****p < 0.0001).

## Supplementary information

Supplementary information

Supplementary Movie1

Supplementary Movie2

Supplementary Movie3

Supplementary Movie4

Supplementary Movie5

## Data Availability

The data that support the findings of this study are available from the corresponding author upon reasonable request.

## References

[1] Wu, A.-M. et al. Global, regional, and national burden of bone fractures in 204 countries and territories, 1990-2019: a systematic analysis from the Global Burden of Disease Study 2019. *Lancet Healthy Longev.* **2**, e580–e592 (2021). 34723233 10.1016/S2666-7568(21)00172-0 PMC8547262

[2] Curtis, E. M. et al. Epidemiology of fractures in the United Kingdom 1988–2012: Variation with age, sex, geography, ethnicity and socioeconomic status. *Bone* **87**, 19–26 (2016). 26968752 10.1016/j.bone.2016.03.006 PMC4890652

[3] Ma, T.-L. et al. Focusing on OB-OC-MΦ Axis and miR-23a to explore the pathogenesis and treatment strategy of osteoporosis. *Front. Endocrinol.*10.3389/fendo.2022.891313 (2022). 10.3389/fendo.2022.891313 PMC9329542 35909545

[4] Ru, J. Y. & Wang, Y. F. Osteocyte apoptosis: the roles and key molecular mechanisms in resorption-related bone diseases. *Cell Death Dis* **11**, 846 (2020). 33046704 10.1038/s41419-020-03059-8 PMC7552426

[5] Khosla, S. & Hofbauer, L. C. Osteoporosis treatment: recent developments and ongoing challenges. *Lancet Diab. Endocrinol.* **5**, 898–907 (2017). 10.1016/S2213-8587(17)30188-2 PMC5798872 28689769

[6] Huang, X. L. et al. Zoledronic acid inhibits osteoclast differentiation and function through the regulation of NF-κB and JNK signalling pathways. *Int. J. Mol. Med.* **44**, 582–592 (2019). 31173157 10.3892/ijmm.2019.4207 PMC6605660

[7] Miller, P. D., Jamal, S. A., Evenepoel, P., Eastell, R. & Boonen, S. Renal safety in patients treated with bisphosphonates for osteoporosis: a review. *J. Bone Miner. Res.* **28**, 2049–2059 (2013). 23907861 10.1002/jbmr.2058

[8] Thangavelu, T., Johnson-Rabbett, B., Magar, R. R. & Khowaja, A. Severe systemic inflammatory response syndrome with multi-organ failure following zoledronic ACID Infusion. *AACE Clin. Case Rep.* **4**, 26–29 (2018).

[9] Cassat, J. E. et al. A secreted bacterial protease tailors the *Staphylococcus aureus* virulence repertoire to modulate bone remodeling during osteomyelitis. *Cell Host Microbe* **13**, 759–772 (2013). 23768499 10.1016/j.chom.2013.05.003 PMC3721972

[10] Qu, S. et al. Rutin attenuates vancomycin-induced renal tubular cell apoptosis via suppression of apoptosis, mitochondrial dysfunction, and oxidative stress. *Phytother. Res.* **33**, 2056–2063 (2019). 31209949 10.1002/ptr.6391

[11] Rybak, M. J. et al. Therapeutic monitoring of vancomycin for serious methicillin-resistant *Staphylococcus aureus* infections: a revised consensus guideline and review by the American Society of Health-system Pharmacists, the Infectious Diseases Society of America, the Pediatr. *Clin. Infect. Dis.* **71**, 1361–1364 (2020). 32658968 10.1093/cid/ciaa303

[12] Shariati, A. et al. Global prevalence and distribution of vancomycin resistant, vancomycin intermediate and heterogeneously vancomycin intermediate Staphylococcus aureus clinical isolates: a systematic review and meta-analysis. *Sci. Rep.* **10**, 12689 (2020). 32728110 10.1038/s41598-020-69058-z PMC7391782

[13] Vallorz, E. L., Encinas-Basurto, D., Schnellmann, R. G. & Mansour, H. M. Design, development, physicochemical characterization, and in vitro drug release of formoterol PEGylated PLGA polymeric nanoparticles. *Pharmaceutics* **14**, 638 (2022). 35336011 10.3390/pharmaceutics14030638 PMC8955426

[14] Zahid, A. A. et al. Cell membrane-derived nanoparticles as biomimetic nanotherapeutics to alleviate fatty liver disease. *ACS Appl. Mater. Interfaces* **16**, 39117–39128 (2024). 39022877 10.1021/acsami.4c08240

[15] Zahid, A. A., Chakraborty, A., Luo, W., Coyle, A. & Paul, A. Tailoring the inherent properties of biobased nanoparticles for nanomedicine. *ACS Biomater. Sci. Eng.* **9**, 3972–3986 (2023). 37378614 10.1021/acsbiomaterials.3c00364

[16] Dykman, L., Khlebtsov, B. & Khlebtsov, N. Drug delivery using gold nanoparticles. *Adv. Drug Deliv. Rev.* **216**, 115481 (2025). 39617254 10.1016/j.addr.2024.115481

[17] Luo, J. et al. The application prospect of metal/metal oxide nanoparticles in the treatment of osteoarthritis. *Naunyn-Schmiedeberg’s. Arch. Pharmacol.* **394**, 1991–2002 (2021). 34415355 10.1007/s00210-021-02131-0 PMC8486704

[18] Chen, L. et al. Pharmaceutical applications of framework nucleic acids. *Acta Pharmaceut. Sin. B* **12**, 76–91 (2022). 10.1016/j.apsb.2021.05.022 PMC8799870 35127373

[19] Fan, Q., Sun, B. & Chao, J. Advancements in engineering tetrahedral framework nucleic acids for biomedical innovations. *Small Methods*10.1002/smtd.202401360 (2024). 10.1002/smtd.202401360 39487613

[20] Huang, J., Chakraborty, A., Tadepalli, L. S. & Paul, A. Adoption of a Tetrahedral DNA Nanostructure as a Multifunctional Biomaterial for Drug Delivery. *ACS Pharmacol. Transl. Sci.* **7**, 2204–2214 (2024). 39144555 10.1021/acsptsci.4c00308 PMC11320733

[21] Sun, Y. et al. Tetrahedral framework nucleic acids loading ampicillin improve the drug susceptibility against methicillin-resistant *Staphylococcus aureus*. *ACS Appl. Mater. Interfaces* **12**, 36957–36966 (2020). 32814381 10.1021/acsami.0c11249

[22] Zhou, M. et al. Effect of tetrahedral DNA nanostructures on proliferation and osteogenic differentiation of human periodontal ligament stem cells. *Cell Prolif.* **52**, e12566 (2019). 30883969 10.1111/cpr.12566 PMC6536416

[23] Cai, L. et al. In silico screening of natural flavonoids against 3-chymotrypsin-like protease of SARS-CoV-2 using machine learning and molecular modeling. *Molecules* **28**, 8034 (2023). 38138524 10.3390/molecules28248034 PMC10745665

[24] Giano, M. C. et al. Injectable bioadhesive hydrogels with innate antibacterial properties. *Nat. Commun.* **5**, 4095 (2014). 24958189 10.1038/ncomms5095 PMC4096704

[25] Kettle, J. K., Grace, J. W., Schaefer, R. S. & Desai, A. Treatment of methicillin-resistant *Staphylococcus aureus* with a vancomycin minimum inhibitory concentration of 2 mcg/mL. *Hospital Pharm.* **45**, 375–380 (2010).

[26] Lin, S. et al. Antioxidative and angiogenesis-promoting effects of tetrahedral framework nucleic acids in diabetic wound healing with activation of the Akt/Nrf2/HO-1 pathway. *ACS Appl. Mater. Interfaces* **12**, 11397–11408 (2020). 32083455 10.1021/acsami.0c00874

[27] Zhu, J. et al. Tetrahedral framework nucleic acids promote scarless healing of cutaneous wounds via the AKT-signaling pathway. *Signal Transduct. Target. Ther.*10.1038/s41392-020-0173-3 (2020). 10.1038/s41392-020-0173-3 PMC7366912 32678073

[28] Fan, Q. et al. Inhalable pH-responsive DNA tetrahedron nanoplatform for boosting anti-tumor immune responses against metastatic lung cancer. *Biomaterials* **301**, 122283 (2023). 37639977 10.1016/j.biomaterials.2023.122283

[29] Zhang, G. et al. Tetrahedral framework nucleic acids as an efficient nasal-to-brain delivery carrier via neural transport pathways. *Adv. Funct. Mater.*10.1002/adfm.202419914 (2025).

[30] Li, Y. et al. A DNA tetrahedron-based ferroptosis-suppressing nanoparticle: superior delivery of curcumin and alleviation of diabetic osteoporosis. *Bone Res.*10.1038/s41413-024-00319-7 (2024). 10.1038/s41413-024-00319-7 PMC10904802 38424439

[31] McDonald, K., Rodriguez, A. & Muthukrishnan, G. Humanized mouse models of bacterial infections. *Antibiotics* **13**, 640 (2024). 39061322 10.3390/antibiotics13070640 PMC11273811

[32] Ravi, S. P. et al. Controlling differentiation of adult stem cells via cell-derived nanoparticles: implications in bone repair. *ACS Appl. Nano Mater.* **5**, 17468–17475 (2022).

[33] Chandrabalan, S. et al. A novel method to efficiently differentiate human osteoclasts from blood-derived monocytes. *Biol. Proced. Online* **26**, 7 (2024). 38504200 10.1186/s12575-024-00233-6 PMC10949786

[34] Shamiya, Y. et al. Ascorbyl palmitate nanofiber-reinforced hydrogels for drug delivery in soft tissues. *Commun. Mater.*10.1038/s43246-024-00641-x (2024). 10.1038/s43246-024-00641-x PMC11415299 39309138

[35] Coyle, A. et al. In vitro engineered ECM-incorporated hydrogels for osteochondral tissue repair: a cell-free approach. *Adv. Healthc. Mater.*10.1002/adhm.202402701 (2025). 10.1002/adhm.202402701 PMC11804842 39757463

