# Supplementary material for: Self-assembled DNA nanocarrier-enabled drug delivery for bone remodeling and antimicrobial applications
Source: NPJ Biomed Innov. 2025 Sep 3;2:29. doi: 10.1038/s44385-025-00020-2 (PMC12408339; doi:10.1038/s44385-025-00020-2)
Supplement: Supplementary file 1 — Supplementary information [file 44385_2025_20_MOESM1_ESM.pdf]

Self-Assembled DNA Nanocarriers for Treating Bone Fracture and Surgery-related MRSA Infection

Jiaqi Huang[1], Aishik Chakraborty[1,2], Yasmeen Shamiya[3], Wei Luo[4], Alap Ali Zahid[1], Arghya Paul[1,2,3,4]*

[1]Department of Chemical and Biochemical Engineering, The University of Western Ontario, London, ON N6A 5B9, Canada

[2]Collaborative Specialization in Musculoskeletal Health Research and Bone and Joint Institute, The University of Western Ontario, London, ON N6A 5B9, Canada

[3]Department of Chemistry, The University of Western Ontario, London, ON N6A 5B9, Canada

[4]School of Biomedical Engineering, The University of Western Ontario, London, ON N6A 5B9, Canada

*Corresponding author
E-mail: arghya.paul@uwo.ca

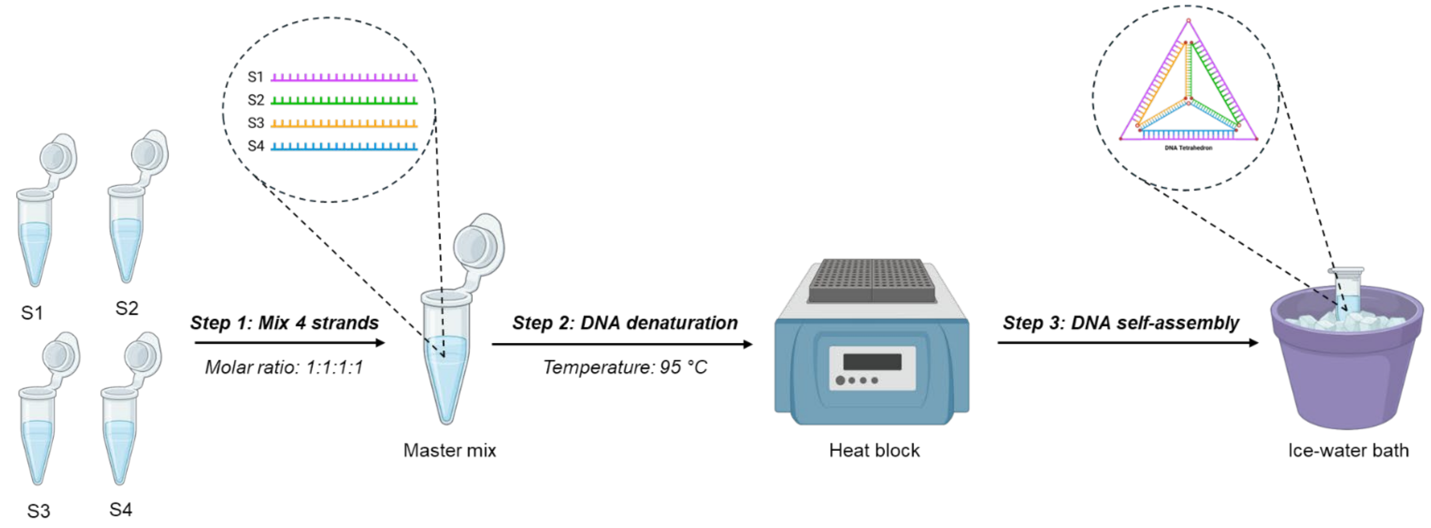

Supplementary Figure 1 | Schematic diagram showing the laboratory procedures of synthesizing TDN.

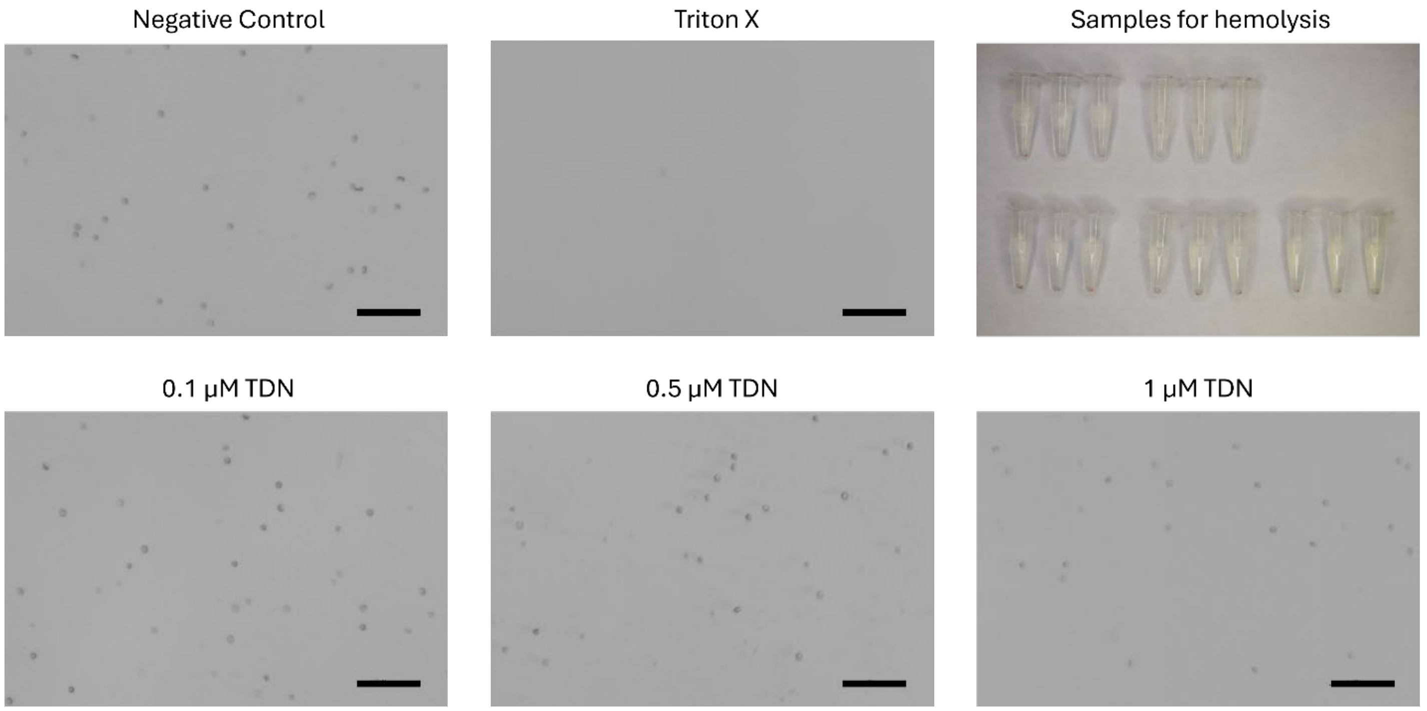

Supplementary Figure 2 | Phase-contrast images of mRBCs following the incubation with PBS (negative control), Triton-X 100 (positive control), 0.1 μm TDN, 0.5 μm TDN, 1 μm TDN. Scale bar 50 μm.

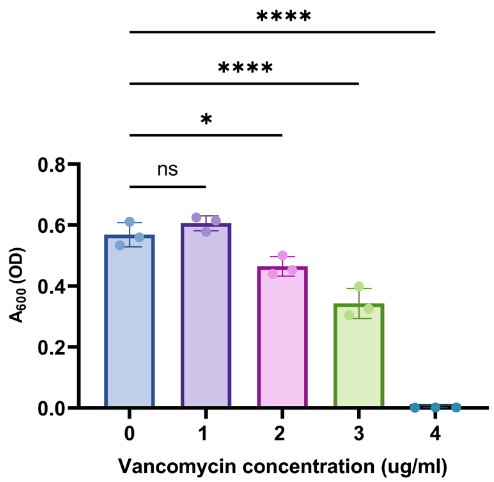

Supplementary Figure 3 | MIC test of vancomycin against MRSA.

Supplementary Movie 1 | Spraying of TDN-integrated hydrogel

Supplementary Movie 2 | Injection of TDN-integrated hydrogel

Supplementary Movie 3 | *In situ* gelation by visible light crosslinking

Supplementary Movie 4 | Examination of sprayed hydrogel

Supplementary Movie 5 | Examination of injected hydrogel
